# Assessment of Binary Agarose–Carbopol Buccal Gels for Mucoadhesive Drug Delivery: Ex Vivo and In Vivo Characterization

**DOI:** 10.3390/molecules27207004

**Published:** 2022-10-18

**Authors:** Muhammad Ali Syed, Sana Hanif, Noor ul Ain, Haroon Khalid Syed, Ameer Fawad Zahoor, Ikram Ullah Khan, Walaa A. Abualsunun, Abdulmajeed M. Jali, Safa H. Qahl, Muhammad H. Sultan, Osama A. Madkhali, Rayan A. Ahmed, Nasir Abbas, Amjad Hussain, Muhammad Abdul Qayyum, Muhammad Irfan

**Affiliations:** 1 Department of Pharmaceutics, Faculty of Pharmaceutical Sciences, Government College University Faisalabad, Faisalabad 38000, Pakistan or; 2 Faculty of Pharmacy, The University of Lahore, Lahore 54590, Pakistan or; 3 Department of Medicine, Fatima Jinnah Medical University Lahore, Lahore 54000, Pakistan; 4 Department of Chemistry, Government College University Faisalabad, Faisalabad 54590, Pakistan; 5 Department of Pharmaceutics, Faculty of Pharmacy, King Abdulaziz University, Jeddah 21589, Saudi Arabia; 6 Department of Pharmacology and Toxicology, College of Pharmacy, Jazan University, P.O. Box 114, Jazan 45142, Saudi Arabia; 7 Department of Biology, College of Science, University of Jeddah, P.O. Box 80327, Jeddah 21589, Saudi Arabia; 8 Department of Pharmaceutics, College of Pharmacy, Jazan University, Jazan 45142, Saudi Arabia; 9 University College of Pharmacy, University of The Punjab, Lahore 38000, Pakistan; 10 Department of Chemistry, Division of Science & Technology, University of Education, Lahore 5600, Pakistan

**Keywords:** marine biopolymer, buccal mucoadhesion, agarose, benzocaine, stability study, in vivo evaluation, oral health care, dental

## Abstract

Agarose (AG) is a naturally occurring biocompatible marine seaweed extract that is converted to hydrocolloid gel in hot water with notable gel strength. Currently, its mucoadhesion properties have not been fully explored. Therefore, the main aim of this study was to evaluate the mucoadhesive potential of AG binary dispersions in combination with Carbopol 934P (CP) as mucoadhesive gel preparations. The gels fabricated via homogenization were evaluated for ex vivo mucoadhesion, swelling index (SI), dissolution and stability studies. The mucoadhesive properties of AG were concentration dependent and it was improved by the addition of CP. Maximum mucoadhesive strength (MS) (27.03 g), mucoadhesive flow time (FT) (192.2 min), mucoadhesive time in volunteers (MT) (203.2 min) and SI (23.6% at 4 h) were observed with formulation F9. The mucoadhesive time investigated in volunteers (MT) was influenced by AG concentration and was greater than corresponding FT values. Formulations containing 0.3%, *w*/*v* AG (F3 and F9) were able to sustain the release (~99%) for both drugs till 3 h. The optimized formulation (F9) did not evoke any inflammation, irritation or pain in the buccal cavity of healthy volunteers and was also stable up to 6 months. Therefore, AG could be considered a natural and potential polymer with profound mucoadhesive properties to deliver drugs through the mucosal route.

## 1. Introduction

Smart gels (SGs) are polymeric dispersions that are capable of altering rheological properties under the external influence of stimuli. Normally, SGs do not change their property but as soon as they are exposed to a stimulus (e.g., moisture), it induces functional changes in the polymeric gel structure, for example, swelling up. The mechanism of these SGs is dependent upon the physicochemical properties of polymers, which respond to the changing conditions [1]. SGs have established biomedical as well as pharmaceutical applications, including tissue scaffold, contact lenses and hydrogels for therapeutic drug delivery [1,2,3,4]. Mucoadhesive delivery is considered to be the extensive application of SGs [5]. Over decades, researchers have worked to develop mucoadhesive polymers from natural or synthetic origins [6,7]. Natural polymers are generally superior to synthetic ones for being biodegradable or biocompatible [8,9,10]. Agarose (AG) is a marine hydrocolloid present normally in red and brown sea weeds possessing biocompatible and biodegradable properties [11]. Agarose has potential biomedical, pharmaceutical, analytical and food applications [1,12,13,14]. When AG is dissolved in hot water, it is converted to a hydrocolloid gel possessing notable gel strength. However, AG gel and adhesive properties were extensively explored for in vitro and in vivo evaluation [15]. When AG was formulated and characterized as mucoadhesive buccal tablets containing AG and CP, the formulations containing AG alone were unable to depict significant mucoadhesive potential in another study [16]. Therefore, the current study was extended to evaluate the mucoadhesive properties of AG via smart buccal gels. For that purpose, AG was combined with Carbopol^®^ (CP), which is a semisynthetic acrylic gelling agent, in smart gels. CP has a documented profile of mucoadhesion as well as swelling and was widely explored in buccal mucoadhesive delivery [17,18]. For local release, benzocaine (BZN) and tibezonium iodide (TIB) were selected as the model anesthetic and antiseptic agents, respectively [1,16,19,20]. Since, antimicrobial activity is important for oral pathological conditions [21]. Nonetheless, the current study was aimed to explore the buccal mucoadhesion of AG via smart buccal gels.

## 2. Results and Discussion

For the physicochemical characterization of formulated gels, evaluation parameters were set, such as appearance, pH, swelling as well as erosion, mucoadhesive studies (ex vivo as well as volunteer) and drug release. Then, formulation was optimized on the basis of complete drug release and better mucoadhesive properties. Moreover, a stability study, solid state characterization, scanning electron microscopy (SEM), statistical analysis and a study on the adaptability response of gel in volunteers were additionally performed on gels to conclude results.

### 2.1. Physicochemical Characterization of SGs

#### 2.1.1. General Appearance

For physical appearance, the clarity order of SGs was AG > AG-CP > CP. No grittiness, color change or precipitation were visualized for formulated gels while dispersing the ingredients. The formulated CP gels (alone/with AG) were very slightly translucent and that increased as the concentration of CP increased. However, AG gels were almost transparent in appearance [22] and increasing concentrations used in the study did not significantly increase the translucency of the gels.

#### 2.1.2. pH

The pH of all the formulations ranged between 6.81–6.93, which is in accordance with normal physiological pH range (6.2–7.6) of the buccal region [23].

#### 2.1.3. Spreadability

Spreadability is an important characteristic to assess the spreading capabilities of the gel under the influence of facial muscle forces. The dosage form that is placed in between the buccal mucosa and the gingiva is pressed when the patient normally responds with facial expressions, speaking as well as ingesting reflexes [24]. Theoretically, there should be a definite value of spreadability so that the mucoadhesive surface area is exposed to mucosal tissue and the gel is not ingested during the former exercises of patient responses. The spreadability values varied according to the nature and concentration of the polymers (Table 1). Maximum spreadability (208.1%) was observed with F4, which contained the lowest concentration of CP (0.2%). When this concentration was blended with AG (0.2%), the value was reduced to 198.2%. However, the lowest value was observed with F3 (157.3%). Generally, an increasing trend in spreadability was observed with formulations containing CP alone. Conversely, decreasing values were observed for gels with increasing concentrations of AG (alone or combined) containing formulations. Since, AG forms stiffened gels with increasing concentration [19], it could be a possibility that stiffness reduces the spreadability value.

#### 2.1.4. Content Uniformity

The content uniformity for both drugs was found in the range of 96–105%. For BZN, the value was in the range of 96.28% to 102.63% for all formulations. The lowest value (96.28%) was observed with formulation F8. However, for TIB, the value ranged between 98.93–101.52%. This outcome depicted satisfactory uniformity for both drugs.

#### 2.1.5. Swelling Index (SI)

**Figure 1:**
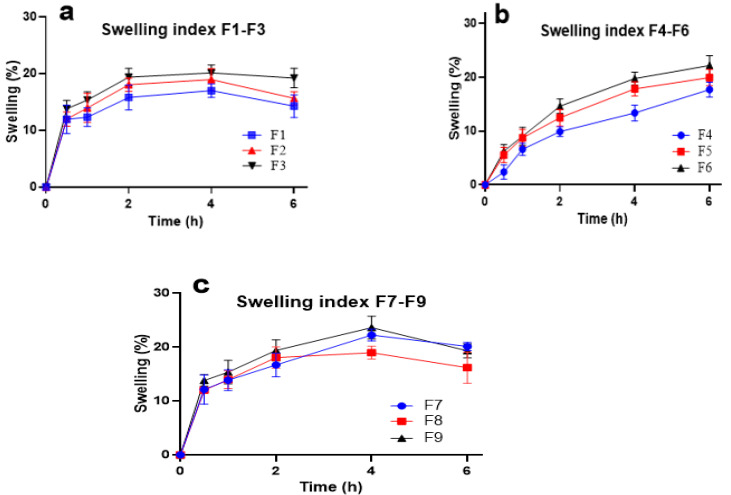
Swellability index of mucoadhesive formulations (**a**). F1–F3 (**b**). F4–F6 and (**c**). F7–F9.

Swellability is an important factor since it is linked with better adhesion to the mucosal surface as well as to release of the drug [25]. A very slight increase in SI was observed as the concentration of polymers in gels increased from 0.2 to 0.4 % (*w*/*v*) [26]. The SI for gels containing CP alone (F4–F6) continued to increase steadily for 6 h (Figure 1a), while it was not observed for AG-based gels until after 6 h. The swellability of AG-based gels reached maximum values of imbibition after 4 h, after which the SI value started declining [17]. As decreasing values of swellability were observed for F3- and F9-based formulations after 4 h, this depicted initial swelling but then slow shrinking after 4 h, probably due to the influx and efflux of PBS [18]. The highest swellability of 23.62% was observed for F9 at 4 h (Figure 1c). The slow swelling of gel was found with CP alone (F4–F6) formulations, F4 being the slowest with 2.38% swelling at 0.5 h. An increasing trend was observed with the SI for F4–F6 in 6 h. In contrast, this trend was not observed after 4 h for AG and AG-CP-based formulations.

#### 2.1.6. Matrix Erosion (ME)

The ME of all formulations was significantly higher (Table 1), which could be due to the lower concentration of polymers. The values of ME were observed to be higher with CP-based formulations (e.g., F6 = 99.71%), and they decreased with the increasing concentration of AG. The lowest ME value was associated with F3 (93.35%), which contained the highest concentration of AG. The addition of CP to AG caused an increase in ME value for AG (Table 1). After ME observations, the AG-based formulations were rehydrated after dryness, thus, showing the gel hysteresis phenomenon, which was unseen in CP [27].

#### 2.1.7. Ex Vivo Mucoadhesive Strength (MS)

**Figure 2:**
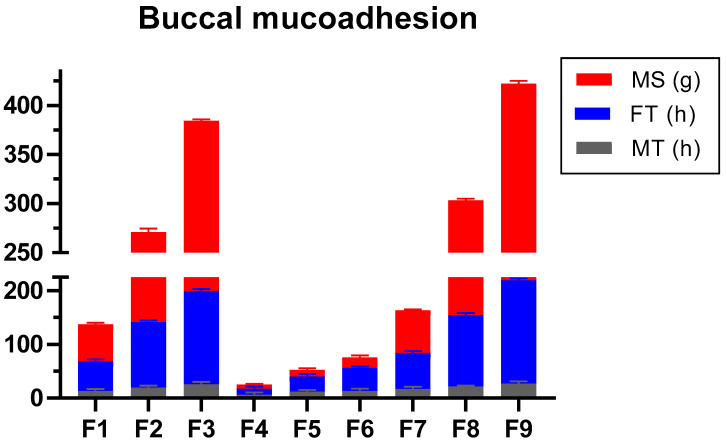
Mucoadhesive parameters of gel formulations.

The results showed that AG formulation possessed better mucoadhesive strength than CP. Poor but concentration dependent MS values were obtained with CP alone formulations (F4–F6). The highest values in the CP-alone-based formulation were seen with F6 (13.60 g). This value was comparable with F1 (13.28 g). However, the value of MS was slightly modified when CP was combined with AG (F7–F9). Therefore, it can be inferred that AG or its blend possessed better MS than CP alone largely due to AG (Figure 2). The MS value is the force required to adhere the dosage to the buccal cavity. Ingredients with poor MS may result in loss of adherence to the buccal mucosa. It may, in turn, cause loss of programmed release at the desirable point. Increased concentration of AG was directly linked to increased MS value. The MS of 0.4% CP (F6) was equivalent to 0.1% AG (F1), which depicted the superiority of AG in gel form. However, the polymeric blend of AG-CP exhibited better MS, which might be due to the intercalation of the hydroxyl group in CP with the hydrophilic groups of AG [28]. Since AG exists as a supercoiled hydrocolloid, it can interact with the COOH and OH groups of CP. Additionally, these functional groups are associated with better hydration and swelling [29,30]. Therefore, it is thought to contribute to improved mucoadhesion.

#### 2.1.8. Ex Vivo Mucoadhesive Flow Time (FT)

Generally, a rise in FT was observed with increasing polymer concentrations (Figure 2). Nevertheless, when the polymers were blended in AC gels, the extent of FT was Ag-CP > AG > CP. F3 had the highest FT of 192.2 min, whereas the values were reduced when AG was dispersed alone, i.e., F9 > F3. The response from AG and its polymeric blend was concentration dependent. It suggests that the findings of FT were associated with polymer nature and concentration similar to MS. The highest values of FT were observed with F9 and F3, which were 192.2 ± 3.56 and 174.0 min ± 3.67, respectively. If FT of F6 is considered, it was quite low (42.8 min) compared to F9 and F3. The FT is an approximation of the time in which dosage forms adhere to the mucosa. Typically, values of MS and FT are linked to each other [8]. Within the concentrations formulated, FT was more linked to the concentration and nature of AG than CP. Since AG is a linear polysaccharide that forms helical fibrillary bundles after cooling in the aqueous solution, it could be responsible for the concentration dependent FT for AG [31].

#### 2.1.9. Dissolution Study

The dissolution methodology for buccal drug delivery typically uses a United States Pharmacopeia (USP) type II paddle apparatus. However, as buccal gels were formulated, the modified forms of the dissolution conditions were adopted as used by the researchers [32]. In order to ensure the formulation would not be squeezed under the pressure of the gums, the in vitro simulated conditions were set. Moreover, another problem that could be faced was the floating of gels on the surface of the dissolution fluid, which could not mimic dissolution guidelines, according to USP. To overcome these issues, a 100 mm mesh was added over the petri dish [1]. Since the transportation of nanoparticles was not studied, therefore, the cellophane tube method was not used.

**Figure 3:**
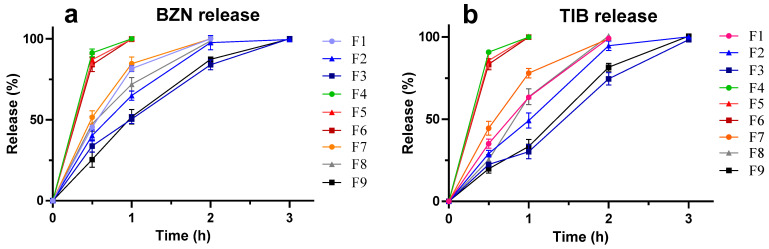
In vitro drug release of smart mucoadhesive buccal formulations for (**a**). BZN and (**b**). TIB.

Using the reported methods, the peaks were identified for BZN and TIB at 2.29 and 4.15 min, respectively, with an initial noise of sodium lauryl sulfate. No peaks for polymers were found to interfere with the peaks of the drugs. The chromatogram is provided as supplementary data. The results revealed that all formulations with different polymeric combinations released BZN within 3 h (Figure 3a). A fast and early release was observed with formulations containing CP alone (F4–F6), which was unable to slow the BZN release. The release of the drug in AG-based formulations was slowed as the concentration of AG in formulations were increased. The release of both drugs was slowed when it was delivered with 0.3% AG (F3), and it released complete BZN within 3 h. More sustainability in F3 was seen at 2 h (83.97%) compared with F2 (97.65%), respectively. At the same concentration, the polymeric blend (F7–F9) released BZN slightly faster compared with formulations containing AG alone (Figure 3a).

The release of TIB, on the other hand, was slightly slower compared with BZN. The CP gels were unable to slowly release the TIB until after 3 h, similar to the releasing trend of BZN. More than 80% of TIB was released within 0.5 h (Figure 3b) for CP. Similarly, AG-based SGs were able to slow down the release of TIB to within 4 h. It could be attributed to the 3-dimensional scaffold of AG [31] that was dependent upon the concentration of AG used. The in vitro release of CP-based (F4–F6) formulations released almost the entire drug within 1 h. However, a more sustainable release of drugs against incremental AG concentration in F3 extended the release from 0.5 to 3 h, possibly due to the increased coiling linear polymer chain of the polymeric nucleus upon gelation [33]. The greater the polymer concentration in the gel, the more the retarding effect of the gel was evident. Because AG is a hydrophilic colloid, it seizes water movement due to the supramolecular colloidal system [34].

#### 2.1.10. Mucoadhesive Time in Healthy Volunteers (MT)

As observed in MS and FT, the outcome of MT also revealed that the formulations containing CP alone exhibited poor residence in the mucosa. As was seen in MS and FT, the highest values were observed with F9 and F3, which were 203.2 and 185.1 min, respectively. The MT of F4–F6 were less than 30 min, which might be due to the lower amounts of polymer used in the study (Figure 2). The lowest value of MT was obtained with F4 (7.2 min). Two volunteers reported the dislocation of AG alone SGs (F1–F3), while it was not recorded with F7–F9. Estimation of MT on the volunteer was performed with formulated SGs without loaded drugs. Toxicity was not a concern since AG is consumed as a food item in different countries worldwide [35]. The human in vivo residence time was correlated with the ex vivo mucoadhesive properties of smart gel composites [36]. In comparison, the rest of the formulations showed a sustainable increase in MT values, and it was proportional to the amount of AG in gel form. Formulations containing the polymeric blend (F7-F9) had a comparatively higher MT value compared to AG alone (F1–F3).

### 2.2. Characterization for Optimized Formulation

#### 2.2.1. FTIR Analysis

**Figure 4:**
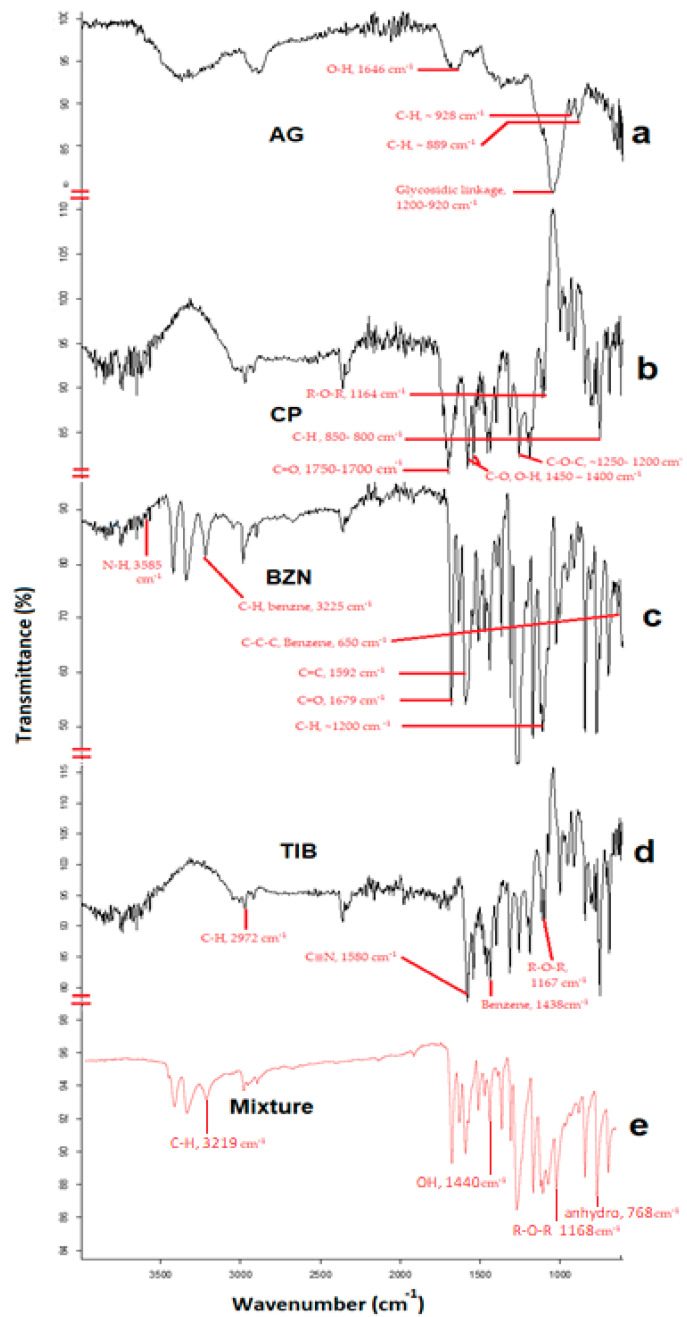
FTIR spectrum of (**a**). AG, (**b**). CP, (**c**). BZN, (**d**). TIB and (**e**). the physical mixture of the drugs and polymers.

The FTIR spectral analysis of CP depicted prominent stretching vibration of the carbonyl group (C=O) between 1750 and 1700 cm^−1^, whereas the peak in the region of 1450–1400 cm^−1^ exhibited the C-O or O-H stretching of the molecule (Figure 4b). The band spectrum ~1250–1200 cm^−1^ depicted the C-O-C of the acrylate derivative. The R-O-R band was shown by the peak around 1164 cm^−1^ indicating its stretching vibration. The peak between 850–800 cm^−1^ represented the C-H out of the plane bending for carbomer [37,38]. In the spectrum for AG, the band at 1646 cm^−1^ corresponded to the bending of the O-H group in the polymer (Figure 4a). The specific absorption band of the polymer, as well as the C-H bending vibrations of the anomeric carbon, were ~928 cm^−1^ and 889 cm^−1^, respectively [39]. The glycosidic linkage in AG was characterized by the stretching vibration of the polysaccharide in between 1200–900 cm^−1^ [40]. For BZN, the C-H stretching vibration was depicted by a sharp minor spectrum at 3225 cm^−1^, whereas the C-C benzene bending was found approximately at 650 cm^−1^. The stretching vibrations in C=O and C=C were observed at approximately 1679 and 1592 cm^−1^, respectively [41]. In the case of TIB, the presence of a cyclic structure was identified with a sharp peak at approximately 1438 cm^−1^ (Figure 4d); however, the major sharp peak at 1580 cm^−1^ was related to C≡N stretching vibrations [42]. Additionally, the FTIR analysis of the physical mixture provided peaks from the components of the drugs and the polymer. It demonstrated an absorption peak at approximately 768 cm^−1^, corresponding to the 3,6-anhydro galactose bending of agarose polymer [43]. The presence of O-H stretching vibration of CP in the mixture was evident in the region of 1440 cm^−1^, which corresponded to similar findings from literature [16,44]. The shift in the -OH vibration to lower values (~3417 cm^−1^) as reported in the literature corresponds to the hydrogen bonding of CP with the hydrophilic groups of AG [45]. Conversely, the C-H vibration in BZN was evident as a sharp peak with a minor shift at approximately 3219 cm^−1^ due to hydrogen bonding. For TIB, the peak at 1593 cm^−1^ was associated with C≡N vibrations. Similarly, the R-O-R stretch around 1168 cm^−1^ was also evident, which endorses the previous findings of the authors in another study [46].

#### 2.2.2. DSC Analysis

**Figure 5:**
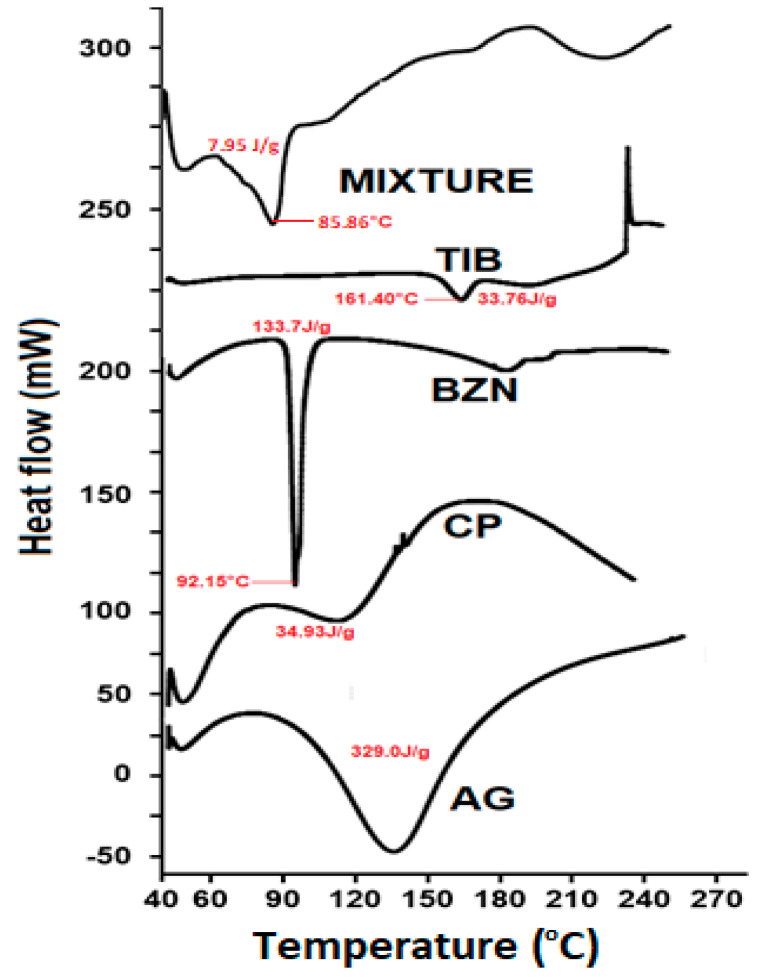
DSC thermogram of AG, CP, TIB, BZN and the physical mixture of the drugs and polymers.

The melting point of pure BZN was identified by a sharp endothermic peak at approximately 92.1 °C (Figure 5), which corresponds to the value reported in the literature [47]. For TIB, melting of the pure drug was confirmed as an endothermic peak depression at 161.3 °C [42]. Likewise, the characteristic endothermic peaks for CP and AG were in accordance with the thermogram found in the literature [48,49,50]. With reference to the endotherm observed for the optimized formulation, no additional or unusual peaks were found in the physical mixture (F9), and the peak was the result of the endothermic behavior of the polymers and drugs. However, a minor shift in the peak of benzocaine was observed to a newer value of 85.86 °C, as reported previously by the authors, but it did not reveal shouldering or an extra peak. The peak of TIB in the physical mixture is present near the melting point of pure TIB, confirming the integrity of the molecule in the mixture (Figure 5). Nevertheless, the peak of TIB was not as sharp compared with the BZN, which is in correlation with the physical mixture, as reported previously [46]. This also demonstrates that the inactive components were non-reactive in the solid state containing benzocaine and tibezonium iodide [51].

#### 2.2.3. X-ray Powder Diffraction (XRD)

**Figure 6:**
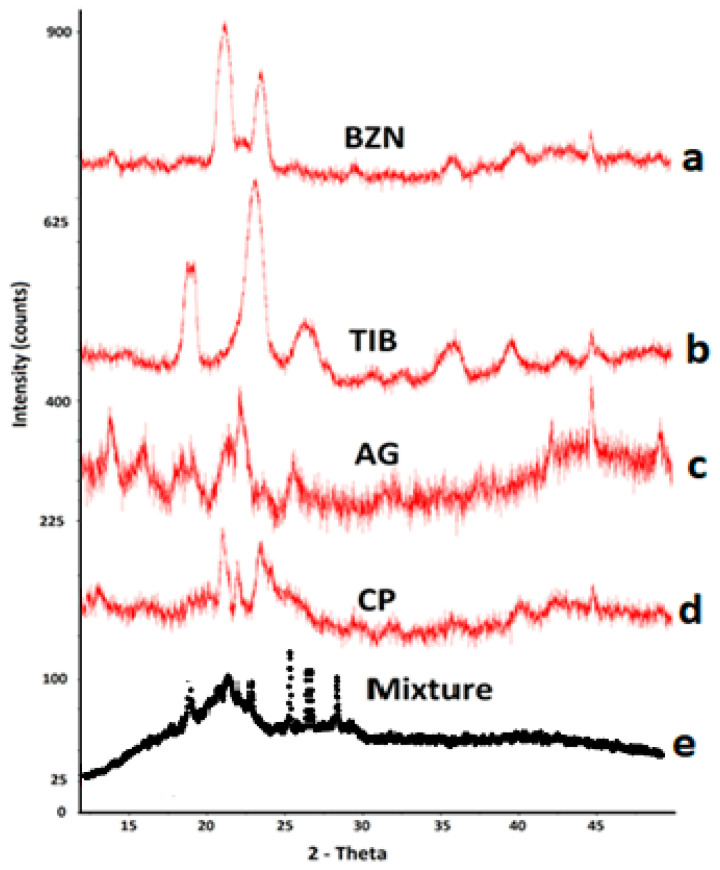
XRD spectra of (**a**) BZN, (**b**) TIB, (**c**) CP, (**d**) AG and (**e**) the physical mixture of polymers with BZN and TIB.

The XRD results of BZN and TIB revealed crystallographic patterns with sharp narrow characteristic peaks (Figure 6). The cluster pattern was distinguishable for drugs in their pure form and was indicative of a crystal pattern [52]. This behavior was also evident with the peaks of CP and AG. The intensity of the peaks for drugs were also observed in the physical mixture [53]. This suggests that no physical change was observed for the drugs in the mixture form. Furthermore, the absence of intense sharp peaks at other points in the XRD pattern depicted that the physical form of the drugs was unaffected; however, due to blending, the signals were reduced. For instance, the sharp peaks pattern of both drugs at 22.3° can be observed as reduced in the physical mixture peak ‘e’ in Figure 6. These findings also are in accordance with the results previously reported by the authors [46].

Based on the results obtained, it was evident that CP alone failed to produce the desirability of sustained release along with significant mucoadhesion compared with AG. However, AG, whether alone or combined, significantly imparted its effects, especially mucoadhesion and sustained drug release. When AG was compared alone or in combination, the polymeric blend exhibited a slightly better response than AG alone. Maximum mucoadhesion was obtained in F9, with the release of both drugs sustained up to 3 h. Based on complete drug release and maximum mucoadhesive character, F9 was chosen as the optimized formulation. It has undergone further evaluation for stability and the in vivo adaptability response in healthy volunteers.

#### 2.2.4. Stability Study

For 6 months, the optimized formulation (F9) was stable in homing both drugs, and at the same time, maintaining the mucoadhesive properties (Table 2). During storage, the gel exhibited acceptable transparency, homogeneity and non-agglomerated character of polymers. The uniformity of contents was also found to be stable for the stated period. For TIB, initially, the concentration of the drug in the gels was almost 100%. However, it was slightly reduced to a value of 97.63%. This slight variation in the amount of TIB was found insignificant with a paired student’s t test as the value of *p* was found to be 0.0728. These finding correlated with the results of Hanif et al. (2022) in a recent finding on a mucoadhesive chitosan-based scaffold [1]. There are studies that support the stability data of polymers used in the current study. For instance, different grades of CP were formulated in a study to deliver meloxicam, and it was found that none of the formulation prepared in the study demonstrated pharmaceutical instability problems during accelerated stability testing [54]. However, there are studies that support that a change in the pH of the CP-based gel formulations during the stability study can impart change in the viscosity of the formulations [55]. Nevertheless, the pH of the optimized formulation in the current study did not change significantly over time (Table 2). Reasonable literature on the stability of agarose-based gel drug delivery is scarce. However, there are reports that when AG was delivered in the presence of antiseptic moieties, then during the stability period, the dosage form was found stable and resistant to deterioration [56].

#### 2.2.5. Statistical Evaluation

Additionally, the in vitro release profiles of both drugs were evaluated in the optimized formulation for statistical analysis. The release profile of the drug after the stability conditions was compared to the in vitro dissolution data of the optimized formulation. If the *p* value in the student’s *t*-test is greater than 0.05, it indicates insignificant differences between the means of the release profile of both drugs. Consequently, it depicts that the significant changes did not occur in the formulations when the gels were placed in the stability chamber under stress conditions. As calculated, the *p* value for BZN and TIB were greater than 0.05, indicating the existence of insignificant difference in the release profile of the drugs (Table 3). The small t-value for both drugs depicts that the difference between the mean values after the stability conditions was insignificant. Subsequently, it confirmed that there was no significant difference. Moreover, the statistical test revealed a standard deviation less than 2% [57]. The small value of standard deviation indicates that the difference between the means was small. It signifies that the means of the parameters of the drug were unaffected by stability conditions. Hence, the gels retained its physical form and did not degrade after the stability study.

**Figure 7:**
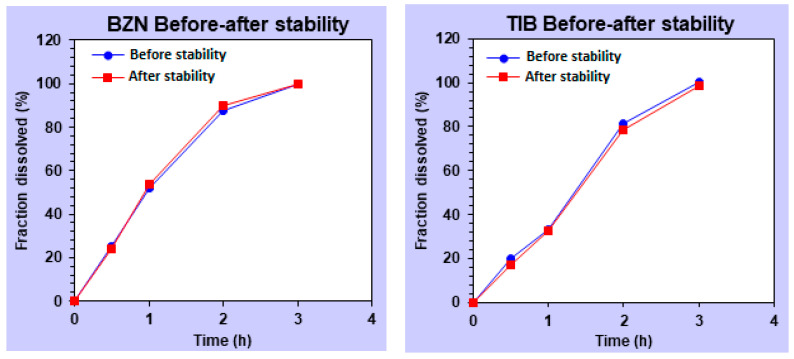
The similarity profile (*f*_2_) of benzocaine and tibezonium iodide after the stability study.

Similarly, for comparison of the release profile, the outcomes of the *f*_1_ factor for both drugs were less than 15 indicating that no significant differences existed between the release profiles for BZN and TIB (Figure 7). Likewise, it was observed that the factor *f*_2_ depicted similarity in between 50 and 100 (Table 4) for both drugs [1].

#### 2.2.6. In Vivo Adaptability Response

Both polymers (AG and CP) possess biodegradability and biocompatibility. AG is consumed as food in different regions of Asia. Therefore, it is supposed to be safer for use in humans. However, dislocation of the dosage form was found in formulations containing CP alone (F4, F5 and F6), which could be attributed to the lower concentration of CP. The volunteers did not report any signs of mouth dryness, inflammation, pain or irritation of the buccal mucosa before 6 h. These findings are in accordance with the previous research in which Carbopol gels were formulated for the local action of metronidazole [6]. This suggests that the gel delivery was adaptable by the volunteers at the concentration used in the study. CP, however, exhibited dislocation of the dosage form within the buccal cavity.

#### 2.2.7. In Vitro Release Kinetics

In vitro release kinetic models were applied to the cumulative release data of both drugs. The model depicting the highest value of coefficient (r^2^) was considered as the best fitted model for each respective drug. The outcome suggested that the drugs followed the Hixson–Crowell mode of drug release [58], as depicted in (Table 5). The values were found to be 0.9941 and 0.9696 for BZN and TIB, respectively. It meant that the release of the drugs was dependent upon the eroding gel in such a way that the surface area was proportional to the cube root of its volume of gel. The mode of release kinetics from the dosage form revealed the Hixson–Crowell model for both drugs, which explains that the erosion of the gel causes the release of drugs from the constantly changing and exposed surface of the gel. A study on the swellability behavior of Carbopol depicted that CP exhibited a porous scaffold at approximately pH 7, while at a pH of approximately 6 and 8, it revealed nodular or thin walled structural forms [59]. When the pH was increased from 6 to 7, interstitial voids began to form between the polymeric gel networks. This affirms that a space should be provided for the drugs to reside in an AG-CP scaffold in formulation F9 that can load BZN and TIB inside gels.

#### 2.2.8. SEM Analysis

**Figure 8:**
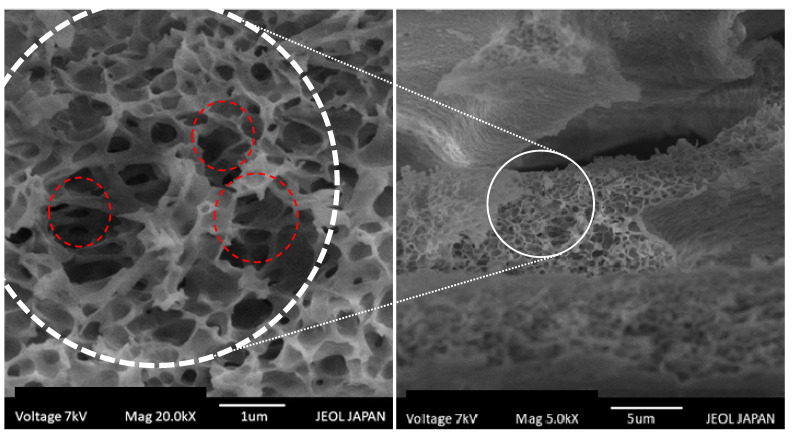
SEM images of smart gel formulation (F9) depicting the Agarose–Carbopol scaffold structure.

The SEM images of the optimized formulation depict the organization of the interconnected porous structure that can be depicted as a scaffold composed of AG-CP in the gel (Figure 8). It was found in a study that when CP was prepared as hydrogels, it exhibited a scaffold structure at approximately pH 7, and this arrangement of gel network did not erode as long as the hydrogels were swollen [59]. Therefore, it was presumed that both loaded drugs were released in the dissolution fluid from the pores of the scaffold (Figure 8). Eventually, it was responsible for the release of the drug in the dissolution media over the period.

## 3. Materials and Methods

### 3.1. Materials

Tibezonium iodide (Recordati^®^) was obtained as a gift from Pacific^®^ Pharmaceuticals Ltd., Lahore, Pakistan. Similarly, Carbopol 934P and benzocaine were generously donated by Remington Pharma, Pvt. Ltd., Lahore, Pakistan. Agarose (labelled gel strength > 1200 g/cm^2^ and a gelling temperature between 35–37 °C) was purchased from bioWORLD^®^ (Dublin, OH, USA). Other solvents/reagents, such as sodium lauryl sulphate (SLS), sodium dihydrogen phosphate, acetonitrile, dimethyl sulfoxide (DMSO), triethanolamine (TEA) and o-phosphoric acid, were of analytical grade and were used as received. For sample filtration, Millipore^®^ was used, while double reverse osmosis (RO) water was used throughout the study unless otherwise specified.

### 3.2. Formulation of Gels

**Figure 9:**
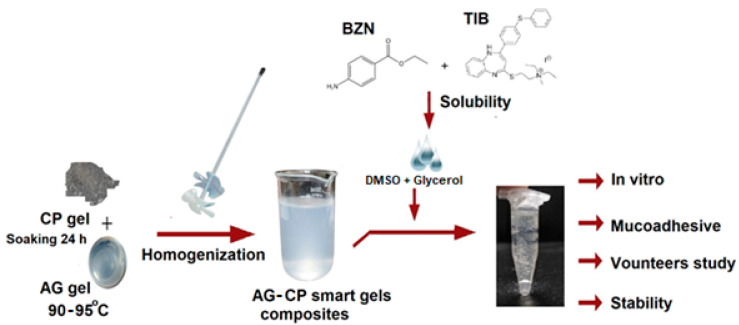
Schematic procedure for the preparation of SGs loaded with drugs.

An accurately weighed amount of AG powder was dissolved at 95 °C in 100 mL distilled water previously containing 0.05 % *w*/*v* of propyl paraben. Afterwards, the solution was cooled to form gels below 40 °C. For the CP gels, weighed amounts of CP were soaked in distilled water previously containing 0.05 % *w*/*v* of propyl paraben (Figure 9). To remove particle clumping [28], the dispersion was homogenized at 1000 rpm for 10 min using a mini basic lab scale Qiangzhong^®^ homogenizer to formulate gels [33]. For formulations containing the polymeric blend of AG and CP (F7–F9), equal masses (Table 6) of formulated SGs of AG and CP were aggressively dispersed for 10 min to form a homogenous dispersion. Then, 0.35 mL (approximately 11.67% *w*/*v* in gel) of DMSO containing the dissolved drugs was added per 3 g of the gel formulation. Finally, 0.15 mL glycerol was added to the dispersion and mixed for 5 min. The pH was set in the range of 6.7–7.0 [23] with triethanolamine. Optionally, drugs were not added to gels when evaluated for mucoadhesive time in healthy volunteers. Otherwise, 5 mg each of BZN and TIB were first dissolved in DMSO and eventually added to gel portions, thereby forming drug loaded SGs [1].

### 3.3. Physicochemical Characterization of Formulated Gels

#### 3.3.1. General Appearance

All formulated mucoadhesive buccal SGs were evaluated for physical appearance in terms of clarity, grittiness, brittleness and color [60].

#### 3.3.2. pH

For pH estimation, the sample of each gel formulation was placed in a small petri dish. The electrode of a digital pH meter was inserted almost 3 mm inside the formulation and the value was noted after stabilization [17].

#### 3.3.3. Spreadability

Spreadability was performed on a large petri dish. Briefly, 0.5 g of the sample was applied inside the pre-marked 1 cm diameter (*D*1) of the circle on a clean glass surface in such a way that the gel did not initially cross the circumference. Then, a mass of 500 g was added when the sample was sandwiched between two glass surfaces for 5 min [61]. It evoked the spreadability of the gel outside *D*1 to a new value (*D*2). Then, the extent of spreadability was calculated using Equation (1).

(1) $$Spreadibility\;\left(\%\right)=\frac{D2}{D1}\times 100$$

#### 3.3.4. Content Uniformity

In order to estimate the content uniformity of SGs, the sample equivalent to a single dose of BZN and TIB was placed in a pestle and mortar. It was then triturated with some volume of dissolution fluid in order to disrupt the three-dimensional structure of the SGs. Furthermore, the intermediate disrupted SGs were shifted and spun in the beaker at 800 rpm with the remaining fluid to make up 900 mL for 45 min at 37.5 °C. Eventually, 5 mL of the sample was removed using a sterile Millipore^®^ syringe filter (0.22 µm) and analyzed for quantitative determination of drugs using the experimental HPLC conditions detailed under the dissolution study [19].

#### 3.3.5. Swelling Index (SI)

An accurately weighed (*W*1) 3 g sample of the formulation was placed on a glass slide, which was then immersed in separate petri dishes containing 10 mL of 6.8 pH phosphate buffer solution (PBS) maintained at 37.5 °C. The glass slide was removed from the respective petri dish and was weighed at defined intervals to estimate swellability. The gain in weight (*W*2) was expressed as *SI* (%) at time ‘t’ for the respective buccal formulation using Equation (2) [62].

(2) $$SI=\frac{W2-W1}{W1}\times 100\;\;\;\;\;\;\;$$

#### 3.3.6. Matrix Erosion (ME)

For *ME*, the swelled formulations were exposed to hot air in a dry oven at 60 °C for 24 h to remove moisture from the swollen SGs. The moisture lost by the gels during the exposure to hot air was evaluated by reweighing (*W*3) the gels after heating. The *ME* was then estimated (Equation (3)) as follows [63].

(3) $$ME\;\left(\%\right)=\frac{W3-W1}{W1}\times 100\;\;\;\;\;$$

#### 3.3.7. Ex Vivo Mucoadhesive Strength (MS)

**Figure 10:**
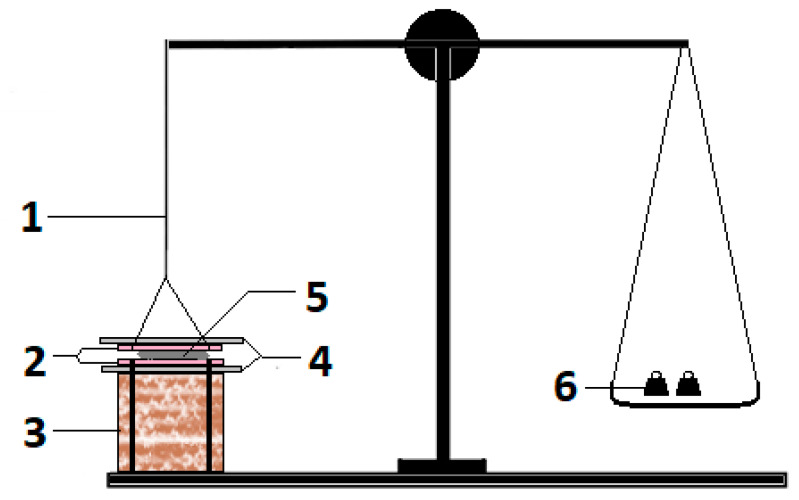
Modified physical balance for estimating ex vivo mucoadhesive strength of buccal gels [65], where 1 = arm replaced with thread, 2 = adhesive buccal mucosa on fixed and movable glass slides, 3 = fixed base, 4 = upper movable (with thread) and lower fixed (with base) glass slide, 5 = gel sample, and 6 = weights added to estimate mucoadhesive strength.

The MS was determined using a modified physical balance using freshly excised buccal mucosa of rabbits as reported by Hanif et al. (2021). Concisely, one arm of balance was replaced with a base on which a slide was fixed (Figure 10). The buccal mucosa was attached on the slides facing the SGs. The gel to be evaluated for MS was placed on the slide and it was covered with another movable slide that was tied to the arm of the balance through the thread. The gel was sandwiched between the moving and fixed slides. When the whole system was static and stable, weight on the left pan was added as drops of water to generate tension on the thread. As the tension increased, the force at which the gel was detached from either surface of the buccal mucosa was considered the MS value [64,65].

#### 3.3.8. Ex Vivo Mucoadhesive Flow Time (FT)

**Figure 11:**
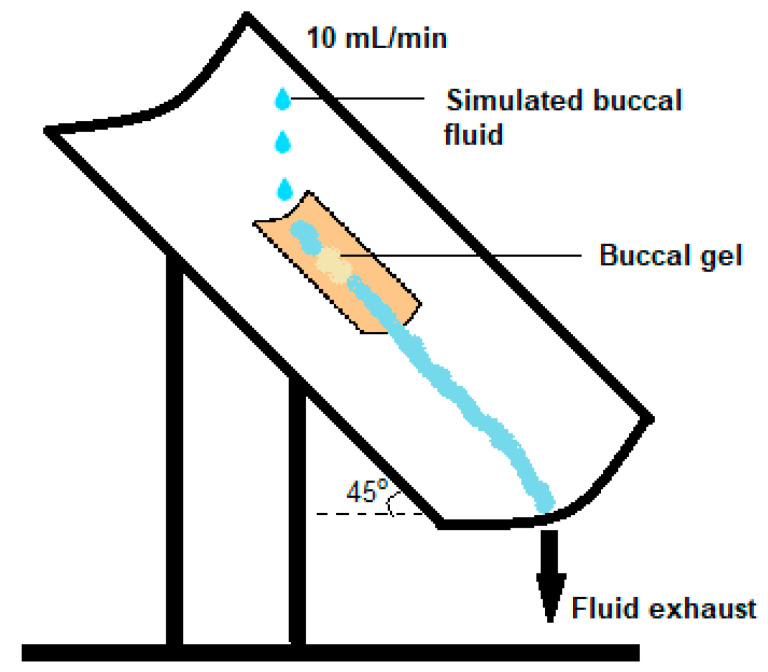
Simulation for estimation of ex vivo mucoadhesive flow time of buccal SGs [27].

The FT for prepared buccal gels was also evaluated using a modified apparatus [26]. Briefly, the longitudinal half of the polyvinyl chloride pipe was inclined at an approximate angle of 60° on which a freshly excised rabbit’s buccal mucosa was adhered with acrylate gum. For FT determination, the formulation was placed on the surface of the mucosa and was kept undisturbed for 20 s to develop mucoadhesion. From the above side of inclined setup, a consistent flow rate of 10 mL/min of PBS adjusted to pH 6.8 was added drop wise on the SGs (Figure 11). The temperature of the PBS was kept at 37.5 °C. The time in which the gel was wiped off the mucosal surface completely was considered as the FT for the respective formulation.

#### 3.3.9. Dissolution Study

The in vitro release studies were performed using a USP type II paddle apparatus for the dissolution of BZN and TIB from mucoadhesive gels. Briefly, 3 g of the gel equivalent to the single doses of BZN and TIB were placed in a watch glass covered with a 100 mm mesh [1,32]. It was then placed at the bottom of the dissolution apparatus containing 900 mL of 0.25% *w*/*v* SLS (adjusted to pH 6.8) solution as the media [1]. The whole set up was maintained at 37.5 °C and a rotation speed of 50 rpm during the experiment. Then, aliquots of 5 mL were removed from the media at defined intervals from 0.5–3 h. The sample was filtered and directly analyzed on an auto injector Agilent^®^ 1260 Infinity (Santa Clara, CA, USA) HPLC machine for the quantitative estimation of both drugs.

#### 3.3.10. HPLC Instrumental Settings

Concisely, the mobile phase was based on acetonitrile and potassium dihydrogen phosphate in a ratio of 70:30 (*v*/*v*) adjusted to pH 4.5 using o-phosphoric acid. The mobile phase was degassed before running in the column. The elution of both drugs out of the column was performed on a C_18_ Agilent^®^ (150 × 4.6 mm, 5 µm) column maintained at 35 °C during the sample runs [19]. A volume of 10 µL was auto injected for each analytical run and detected at 318 nm.

#### 3.3.11. Mucoadhesive Study in Volunteers (MT)

Volunteers (m/f, 20–27 years old) willing to participate were included. Food was prohibited to consume during the experiment, while a liquid diet was not barred, however, rinsing the buccal cavity with the liquid was not allowed. The protocols for determination of MT were followed [36,57]. Formulated drug-free buccal SGs were applied gently in between the lower gum and the inferior labial frenulum [66].

### 3.4. Characterization for Optimized Formulation

#### 3.4.1. Fourier Transform Infrared (FTIR)

The FTIR analysis was performed on the samples of TIB, BZN, CP, AG and the physical mixture as used in the formulation. Approximately 10 mg of the powder sample was directly placed on the lens of the machine. The infrared spectra in the range of 4000–600 cm^−1^ were obtained by operating Bruker Alpha™ (operated by OPUS^®^) Platinum-ATR in transmission mode [65].

#### 3.4.2. Differential Scanning Calorimetry (DSC)

DSC analysis was performed on pure drugs and polymers. as well as their physical mixture when combined in equal proportions of the drugs and the polymers. To be precise, each sample weighing approximately 10 mg was placed in an aluminum pan sealed with lids in a Modulated DSC TL^TM^ Q2000 machine. The response of the thermogram was obtained at a rate of 20 °C per minute over a scanning range of 40 to 250 °C using nitrogen as an inert purging gas at a rate of 50 mL/min [42]. The physical mixture was comprised of quantities of both polymers 3 to 4 times greater than the single doses of the drugs.

#### 3.4.3. X-ray Powder Diffraction (XRD)

The samples of pure drugs and their physical mixture, along with the polymers, were evaluated for XRD analysis by exposing them to MiniFlex^®^ 600X-ray diffractometer (Rigaku^®^, Japan) to observe the changes in the physical forms of ingredients. Briefly, the samples were subjected to an incrementing voltage of 40 kV with current in the range of 15 mA. The exposed angle (2-theta) ranged between 5–45° [67]. The physical mixture was comprised of quantities of both polymers 3 to 4 times greater than the single doses of the drugs.

#### 3.4.4. Stability Study

The optimized buccal formulation was subjected to stability storage conditions according to the guidelines of the International Council for Harmonization (ICH). Concisely, the gel was placed in a tight air sealed glass test tube with a plastic lid and adhesive tape over the cap. It was stored at 40 °C with a relative humidity (RH) of 70 % ± 5 for a duration of 6 months in the stability chamber with intermittent sampling points. At intervals, the samples of stored gels were evaluated for physical appearance, drug contents, mucoadhesive strength (MS) and flow time (FT).

#### 3.4.5. Statistical Evaluation

The optimized formulation before and after the stability studies was also calculated for statistical analysis (paired student’s *t*-test), dissimilarity (*f*_1_) and similarity factors (*f*_2_) to analyze whether the difference between the in vitro drug release really exists or not [42].

#### 3.4.6. Volunteer Adaptability Response

The prepared buccal gels without loaded drugs were also evaluated for parameters such as mouth dryness, inflammation, pain or irritation, and dislocation of the dosage form in healthy human volunteers while estimating the MT values.

#### 3.4.7. In Vitro Drug Release Kinetics

In vitro drug release of the optimized formulation was performed to evaluate the mode of BZN and TIB release from the mucoadhesive gel form. Kinetic models such as zero order, first order, Higuchi, Korsmeyer–Peppas and Hixson–Crowell were applied on the in vitro release data of BZN and TIB using a DD^®^ solver [65].

#### 3.4.8. Scanning Electron Microscopic (SEM) Analysis

The SEM analysis was performed on the optimized mucoadhesive buccal formulation to envision the microstructure of the polymeric array in gel form. The formulations were placed on the copper plate and sputtered before imaging using 20.0 kX magnification and 15 kV in scanning electron microscope JEOL^®^, Tokyo, Japan [68].

## 4. Conclusions

The current study depicted that no interaction was found between drugs, polymers and their physical mixture, as was evident from the FTIR and DSC results. The unaffected physical form of drugs in the mixture was confirmed through XRD analysis. It was found that Carbopol alone exhibited a poor response for mucoadhesive strength, mucoadhesive flow time, in vivo residence time and drug release at the investigated concentrations compared to AG-based gels. However, the parameters were improved when gels were blended. Nonetheless, in the blend form, it was found that the mucoadhesive and drug release properties were mainly contributed by AG. The findings of the dissolution study suggested that AG alone (F1–F3) could sustain the release up to 3 h with 0.4% concentration (F6). The formulation F9 exhibited better mucoadhesion as well as slowed the release of drugs in the simulated dissolution fluid. The optimized formulation (F9) was stable at RH of 70 ± 5 % and 40 °C for 6 months and no significant difference in the release profile of benzocaine and tibezonium iodide was observed statistically. In conclusion, AG could be used as a potential mucoadhesive agent to deliver drugs through the mucoadhesive route.

## Figures and Tables

**Table 1:** Physicochemical characterization of smart mucoadhesive buccal gels.

Code	Spreadability (%)	ME (%)
F1	183.1 ± 0.66	96.36 ± 1.20
F2	195.7 ± 1.46	96.62 ± 3.29
F3	157.3 ± 1.37	93.35 ± 2.42
F4	208.1 ± 1.29	98.37 ± 1.44
F5	162.8 ± 0.46	99.44 ± 0.75
F6	173.5 ± 1.03	99.71 ± 2.51
F7	198.2 ± 0.60	98.13 ± 2.14
F8	180.1 ± 0.86	98.01 ± 1.98
F9	177.6 ± 1.18	97.7 ± 1.03

**Table 2:** Evaluation of different physicochemical parameters during stability studies.

Time (Months)	pH	MS (g ± SD)	FT (min ± SD)	Contents (% ± SD)	
				BZN	TIB
0	6.82	27.03 ± 4.19	150.2 ± 3.56	98.01 ± 2.32	100.57 ± 1.42
0.5	6.80	28.16 ± 3.96	155.8 ± 4.32	99.23 ± 1.16	100.25 ± 0.07
1	6.83	27.97 ± 1.77	153.5 ± 2.96	98.29 ± 0.39	97.37 ± 0.10
3	6.78	29.77 ± 2.02	152.2 ± 3.18	99.76 ± 0.37	98.16 ± 1.39
6	6.77	29.97 ± 1.29	155.0 ± 4.14	98.88 ± 0.90	97.63 ± 0.72

**Table 3:** Statistical analysis of the optimized formulation after the stability conditions.

Before—After Stability	Mean	Standard Deviation	Standard Error Mean	95% Confidence Interval of the Difference		t Value	df	Sig. (2-Tailed)
				Lower	Upper			
BZN	0.560	1.593	0.712	−1.418	2.538	0.786	4	0.341
TIB	1.718	1.243	0.556	−3.2621	−0.173	−3.08	4	0.068

**Table 4:** Similarity and similarity index of the optimized formulation undergoing a stability study.

Parameter	Specifications	BZN	TIB
Similarity factor (f_2_)	50–100	86.89	82.12
Dissimilarity factor (f_1_)	0–15	2.22	3.65

**Table 5:** In vitro release kinetics of BZN and TIB from the optimized (F9) formulation.

Model	BZN Release			TIB Release		
	k	r^2^	n	k	r^2^	n
Zero order	30.059	0.7499	-	28.610	0.8829	-
First order	0.795	0.9797	-	0.616	0.9444	-
Higuchi model	52.41	0.9514	-	48.464	0.9137	-
Korsmeyer–Peppas model	51.13	0.9524	0.528	37.74	0.9189	1.02
Hixson–Crowell model	0.224	0.9941	-	0.175	0.9696	-

**Table 6:** Composition of mucoadhesive smart gel formulations (%, *w*/*v*).

Codes	F1	F2	F3	F4	F5	F6	F7	F8	F9
AG (%)	0.2	0.3	0.4	-	-	-	0.2	0.3	0.4
CP (%)	-	-	-	0.2	0.3	0.4	0.2	0.3	0.4

## Data Availability

The authors confirm that data are contained within the article.

## Sample Availability

Samples of the compounds will be available on demand.
